# Subcutaneous dissociative conscious sedation (sDCS) an alternative method for airway regional blocks: a new approach

**DOI:** 10.1186/1471-2253-11-19

**Published:** 2011-10-26

**Authors:** Mihan J Javid

**Affiliations:** 1 Department of Anesthesiology, Imam Khomeinee Medical center, Tehran University of Medical Sciences, Iran

## Abstract

**Background:**

Predicted difficult airway is a definite indication for awake intubation and spontaneous ventilation. Airway regional blocks which are commonly used to facilitate awake intubation are sometimes impossible or forbidden. On the other hand deep sedation could be life threatening in the case of compromised airway.

The aim of this study is evaluating "Subcutaneous Dissociative Conscious Sedation" (sDCS) as an alternative method to airway regional blocks for awake intubation.

**Methods:**

In this prospective, non-randomized study, 30 patients with predicted difficult airway (laryngeal tumors), who were scheduled for direct laryngoscopic biopsy (DLB), underwent "Subcutaneous Dissociative Conscious Sedation" (sDCS) exerted by intravenous fentanyl 3-4ug/kg and subcutaneous ketamine 0.6-0.7 mg/kg. The tongue and pharynx were anesthetized with lidocaine spray (4%**)**. 10 minutes after a subcutaneous injection of ketamine direct laryngoscopy was performed. Extra doses of fentanyl 50-100 ug were administered if the patient wasn't cooperative enough for laryngoscopy.

Patients were evaluated for hemodynamic stability (heart rate and blood pressure), oxygen saturation (Spo_2_), patient cooperation (obedient to open the mouth for laryngoscopy and the number of tries for laryngoscopy), patient comfort (remaining moveless), hallucination, nystagmus and salivation (need for aspiration before laryngoscopy).

**Results:**

Direct laryngoscopy was performed successfully in all patients. One patient needed extra fentanyl and then laryngoscopy was performed successfully on the second try. All patients were cooperative enough during laryngoscopy. Hemodynamic changes more than 20% occurred in just one patient. Oxygen desaturation (spo_2_< 90%) didn't occur in any patient.

**Conclusions:**

Subcutaneous Dissociative Conscious Sedation (sDCS) as a new approach to airway is an acceptable and safe method for awake intubation and it can be suggested as a noninvasive substitute of low complication rate for regional airway blocks.

**Registration ID in IRCT:**

IRCT201012075333N1

## Background

Difficult airway is defined as difficult ventilation and/or intubation resulting from anatomic or pathologic problems or a situation in which optimal positioning of the patient may be unsafe [1].

Predicted difficult intubation is a definite indication for maintaining spontaneous ventilation and awake intubation and using regional anesthesia in such a situation is advantageous because the patient is able to cooperate with the operator [2]. Airway regional blocks are commonly used for awake intubation in patients with diagnosed compromised airway. The simplicity of these techniques is one of the advantages of airway blocks [3] but sometimes because of the nature of the pathology such as burns or the location of the tumors, airway regional blocks are impossible or unsafe. Laryngopharyngeal lesions are among the common causes of predicted difficult intubation and the airway is very difficult to handle [4]. Patients with laryngeal tumors underwent Direct Laryngoscopic Biopsy (DLB) before their therapeutic planning. Direct laryngoscopic Biopsy which performs through rigid bronchoscope requires endotracheal intubation and general anesthesia, and awake tracheal intubation is the method of choice before induction of general anesthesia.

A variety of methods have been used to provide enough sedation for airway manipulation and awake intubation [5-16] when airway regional blocks are unsafe or contraindicated. Hypoxemia and desaturation because of the respiratory depression have been observed in most of the studies. Dissociative conscious sedation as a method with the characteristics of maintaining spontaneous ventilation, patient cooperation, patient comfort and enough amnesia has been reported in 2011 [17].

The aim of this study is the evaluation of the efficacy and safety of sub-anesthetic doses of subcutaneous ketamine in conjunction with narcotics (subcutaneous dissociative conscious sedation) as an alternative method to airway regional blocks.

## Methods

The protocol was approved by the ethics committee of Tehran University of Medical Sciences and written consent was received from the patients who participated in this study.

30 male patients with the mean age of 62.4 (range 45-81 years) and average body weight of 63.1 kg (range 48-85 kg), classified as ASA class I and II with predicted difficult intubation and no access to landmarks needed for airway regional blocks, scheduled for direct laryngoscopic biopsy (DLB) were enrolled in this prospective, non randomized study between December 2009 and February 2010.

Patients with the history of Psychological disorders, coronary artery disease, uncontrolled hypertension, increased ICP, intracranial mass lesions and open eye injury were excluded from the study.

After the establishment of an intravenous cannula, conscious sedation was exerted by an intravenous injection of fentanyl (3-4 ug/kg) and a subcutaneous injection of ketamine 0.6-0.7 mg/kg (0.5 mg/kg +10 mg). Subcutaneous ketamine was injected on the anterior aspect of the forearm.

The tongue and pharynx were anesthetized with 1-2 ml lidocaine spray (4%).

In order to diminish the pain resulting from subcutaneous injection, fentanyl was injected a few minutes before subcutaneous ketamine.

Patients were monitored continuously using standard monitoring techniques including electrocardiography, pulseoximetry and noninvasive blood pressure. All patients received supportive oxygen by mask before direct laryngoscopy and tracheal intubation.

After 10 minutes, when the desirable level of conscious sedation was achieved, direct laryngoscopy was performed. The desirable level of conscious sedation was defined as an arouseable patient with proper responses to verbal commands. Additional intravenous fentanyl (50-100 ug) was administered if the level of sedation was not appropriate to keep the patient moveless during direct laryngoscopy. The patients were evaluated for hemodynamic stability (heart rate and blood pressure), oxygen saturation (Spo_2_), patient cooperation (obedient to open the mouth for laryngoscopy and the number of tries for laryngoscopy), patient comfort (remaining moveless), hallucination, nystagmus and salivation (need for aspiration before laryngoscopy).

After asking the patients to open their mouth, direct laryngoscopy and tracheal intubation (if possible) were performed.

Narcotic induced respiratory depression, if it occurred, was reversed by asking the patient to breathe.

If the systolic blood pressure increased more than 20% from the baseline and/or exceeded 170 mmHg, TNG 50 μg IV was given in incremental doses (repeated doses of TNG 50 ug) until the systolic blood pressure reached 140 mmHg.

Adverse effects of the method were recorded. Adverse events were defined as irreversible apnea (by routine stimulation), hypoventilation and hypoxemia (Spo_2 _< 90%), laryngospasm, and upper airway obstruction. Serious adverse events were defined as the need for positive pressure ventilation, insertion of an oral or nasal airway, urgent endotracheal intubation or tracheostomy. Minor adverse events were defined as respiratory events requiring minimal intervention (stimulation, supplemental O2 and head repositioning).

It should be kept in mind that in this method, we need an optimum level of sedation before laryngoscopy and tracheal intubation, and then we have to wait 10 minutes after drug administration in order to achieve the desirable level of sedation. When the patient falls asleep (the eyes are closed while there is no stimulation) the patient is ready for laryngoscopy. On the other hand local anesthesia of oropharynx is necessary as well.

## Results

Direct laryngoscopy was performed successfully in all patients. Tracheal intubation was accomplished in 28 patients, and in 2 patients direct laryngoscopy showed large obstructive tumors and impossible tracheal intubation. Tracheostomy was established for these 2 patients. One patient needed extra fentanyl and then laryngoscopy was performed on the second try. All patients were cooperative and obedient during laryngoscopy and intubation. Hemodynamic changes more than 20% occurred in just one patient. Oxygen desaturation (spo_2_< 90%) didn't occur in any patient. Mild to moderate nystagmus was observed in 25 patients a few minutes after ketamine injection. All patients were satisfied with their laryngoscopy. Narcotic induced respiratory depression occurred in two patients and it was easily reversed by asking the patient to breathe. An event of recall was reported but it was not classified as annoying. No salivation was observed. (Table 1)

**Table 1:** subjects' clinical data

P	Complications
Increased BP > 20%	1/30

SPO_2 _< 90%	0

Recall for annoying events	0

Irreversible Apnea (No response to asking the patient to breath)	0

Nystagmus	25

Hallucination	0

Salivation (need for aspiration before lartngoscopy)	0

Laryngospasm and airway obstruction	0

Need for positive pressure ventilation	0

## Discussion

Difficult airway is defined as difficult ventilation and/or intubation resulting from anatomic or pathologic problems or a situation in which optimal positioning of the patient may be unsafe [1].

Airway regional blocks have been commonly used for years in patients with anticipated difficult intubation because of the ability to maintain spontaneous ventilation, airway patency and cooperation of the patient [2].

The simplicity of these techniques is one of the advantages of airway blocks [3] but complications such as elimination of highly effective airway protective reflexes, bleeding, nerve damage and intravascular injection are the disadvantages of airway blocks [2].

Besides the complications mentioned above, the absence of enough access to airway landmarks needed for regional airway blocks in oropharyngeal, laryngeal and neck tumors and obesity, are other encouraging reasons to search for alternative methods to regional airway blocks. Laryngopharyngeal lesions are among the common causes of predicted difficult intubation and the airway is very difficult to handle [4] and therefore airway regional blocks are commonly used in these cases to keep the airway secure during laryngoscopy and intubation. Airway manipulation without sedation is terribly annoying and the anesthesiologist is obliged to use a kind of sedation.

In laryngeal tumors Direct Laryngoscopic Biopsy (DLB) performs through rigid bronchoscope and this kind of surgery requires general anesthesia, and tracheal intubation is necessary in order to prevent aspiration of blood and tumor particles. General anesthesia is induced after awake tracheal intubation

Laryngeal tumors (supraglotic, glotic, subglotic) produce partial mild to severe obstruction of the airway at the glotic or subglotic region (predicted difficult airway) and sometimes localization and/or the bulk of the tumor make the intubation impossible. Therefore we have to keep the patient awake during the laryngoscopy and intubation in order to maintain a secure airway.

In this study "subcutaneous Dissociative Conscious Sedation" was evaluated as a non invasive method for awake laryngoscopic procedures to provide enough sedation and a peaceful situation for the patient by preparing a cooperative, conscious and deeply sedated patient for the anesthesiologist during the laryngoscopy and intubation.

The most important advantage of this method is the ability to maintain spontaneous ventilation in a deeply sedated patient while the patient is cooperative enough to obey.

Conscious sedation as a method to safeguard the patients with compromised airway is a confusing term because we need a fully sedated, calm patient with no respiratory depression and no disturbing effect on the cooperation. A variety of methods have been used to provide enough analgesia and sedation during the manipulation of airway in an awake patient [5-16].

Dissociative conscious sedation was designed and used for the first time in 2004 by the author for laparoscopic implantation of peritoneal dialysis catheter in patients who had very poor physical condition because of their end stage chronic renal failure and related complications (vascular access problems for hemodialysis, severe cardiovascular diseases, volume overload, severe fluid and electrolyte imbalances,...) and were not suitable for general anesthesia [17]. Dissociative conscious sedation or in other words dissociative conscious anesthesia is defined as using an intravenous or subcutaneous injection of "low dose ketamine' in conjunction with narcotics to achieve an acceptable level of sedation, pain relief and amnesia [17]. Then this method was reported as an alternative method for regional airway blocks in "The 1st International Congress of Airway Management/and Anesthesia in Head and Neck Surgery" [18,19]. In the two previous studies a very low dose of midazolam (0.015 mg/kg) was used in order to achieve a desirable level of sedation and amnesia [17,18]. Although using midazolam has been reported in order to improve patient's comfort [20], it can be life threatening in the case of difficult airway because it causes an unintended deep sedation, hypoxemia, desaturation [21] and upper airway obstruction which is irreversible with flumazenil [21,22], also midazolam interferes with the patient's cooperation considerably. Using midazolam in conjunction with other drugs can be dangerous and life threatening in the case of compromised airway and it should be avoided.

Regarding the unique ability of ketamine to provide simultaneous anxiolysis, analgesia and amnesia and maintaining airway and breathing reflexes [23] this study was conducted without using midazolam and patient's comfort and amnesia were provided by using a low dose of ketamine. The study showed that "low dose ketamine" can be an appropriate substitute for midazolam. The dose of ketamine for inducing dissociative conscious sedation can be variable in different procedures (o.5 mg/kg -1 mg/kg).

Ketamine has been used for decades as an analgesic and anesthetic drug. Ketamine is used by oral, intranasal, rectal and subcutaneous routes [24].

In the recent decade, sub anesthetic doses of ketamine have been used as an adjuvant to increase the duration of action and the analgesic effect of narcotics in palliative care, in the control of chronic pain [25] and postoperative pain [26]. Sub-anesthetic dose of ketamine as an adjuvant to narcotics has a dramatic pain relief and opioid dose sparing effect [24]. Ketamine is a small lipophilic molecule with rapid onset and relatively short duration of action (about 15 minutes), in intravenous administration and it requires continuous infusion for maintenance of clinical effects [27].

In this study the subcutaneous route of injection was chosen, because the gradual absorption of the drug decreases the adverse effects because of the low plasma concentration, and increases the duration of the analgesic effect of narcotics considerably. It has been shown that psychomimetic side effects can be prevented by keeping the plasma concentration at or below 150 ng/ml [28] and psychomimetic side effects have not been reported after rectal administration, which is known to result in low plasma levels [29]. A subcutaneous infusion might therefore provide analgesia without hallucinations or other psychotomimetic effects [30,31].

Avoiding midazolam increases the safety of dissociative conscious sedation considerably.

By avoiding midazolam, narcotic related respiratory depression is easily reversible by asking the patient to breathe.

Comparison of subcutaneous and intravenous Dissociative Conscious Sedation [17] showed the superiority of subcutaneous DCS.

The results of this study confirmed that subcutaneous DCS provides an appropriate situation for a safe and successful manipulation of the airway and tracheal intubation, with the ability to maintain a patent airway, spontaneous ventilation, and a deeply sedated, cooperative and obedient patient during the procedure.

### Limitations of the study

This study as an interventional initiative study had some limitations. Because of the ethical limitations for using a new interventional method, I had to include only ASA class I and II patients and choose the patients who had no accessibility to the landmarks needed for airway regional blocks and therefore airway regional blocks were impossible or contraindicated. Cases in this category of difficult airway are not common. Given the limitations mentioned above and as a preliminary study, the small sample size of the study may be excusable.

On the other hand small sample size and high incidence of laryngeal tumors in men (much more common in men) resulted in another limitation and the study included only male patients.

Regarding the limitations mentioned above obviously we need complementary studies in the future. This method should be evaluated in comparison to other pre-existing methods of awake intubation as well.

## Conclusion

Subcutaneous Dissociative Conscious Sedation (sDCS) as a new approach to airway is an acceptable and safe method for awake intubation and it can be suggested as a noninvasive substitute of low complication rate for regional airway blocks.

## List of abbreviations

sDCS: subcutaneous Dissociative Conscious Sedation; DLB: Direct Laryngoscopic Biopsy

## Competing interests

The authors declare that they have no competing interests.

## Authors' contributions

MJJ carried out study designing, data collection and manuscript writing.

## Pre-publication history

The pre-publication history for this paper can be accessed here:

http://www.biomedcentral.com/1471-2253/11/19/prepub
